# IL-17 as an important effector cytokine in a mouse model for *S.typhimurium* sepsis

**DOI:** 10.1186/1479-5876-10-S3-P14

**Published:** 2012-11-28

**Authors:** Andrea Schroll, Manfred Nairz, Igor Theurl, Günther Weiss

**Affiliations:** 1 Univ. Clinic, Dept. of General Internal Medicine, Clinical Immunology and Infectious Diseases, Innsbruck, Austria

## Background

Naïve CD4^+^ helper T cells differentiate after TCR activation into the 3 characterizing cytokine-secreting effector cells: T_H_-1, T_H_-2, and T_H_-17. The T_H_-17 lineage is characterized by IL-17 production and is induced by IL-23, IL-6 and TGF-ß.

The role of iron in IL-17-mediated immune functions however, has not been elucidated thus far. Herein, we investigated the involvement of IL-17 in immune response against *S. typhimurium.*

## Materials

40 C57bl/6 mice were divided into 10 groups. 20 mice received an iron enriched diet, whereas the others were kept on standard diet. After 3 weeks 16 mice of the iron group and 16 mice of the non-iron group were infected 500 CFU (colony forming units) *Salmonella typhimurium* by intraperitoneal injection. In addition, 8 mice fed the iron diet and 8 mice of standard diet mice received 100µg of a blocking IL-17 monoclonal antibody 2 hours prior to infection by intraperitoneal injection. Mice were either killed 6h or 24h post infection and the bacterial load in liver and spleen was quantified by plating organ homogenates.

## Results

IL-17 blockade resulted in an increased weight loss in iron overloaded and control mice. Additionally, spleen weight was upregulated 24h post infection. Intriguingly, we could observe increased CFU in blood, liver and spleen in IL-17 mAb treated 6h and 24h post infection.

## Conclusion

We could show the importance of IL-17 in *S.typhimurium* infection and the combination of iron overload and IL-17 blockade aggravates the severity of *S.typhimurium* infection. Thus, our data suggests that iron might play a crucial role in Th17 immune response.

